# Investigation of Gene Regulatory Networks Associated with Autism Spectrum Disorder Based on MiRNA Expression in China

**DOI:** 10.1371/journal.pone.0129052

**Published:** 2015-06-10

**Authors:** Fengzhen Huang, Zhe Long, Zhao Chen, Jiada Li, Zhengmao Hu, Rong Qiu, Wei Zhuang, Beisha Tang, Kun Xia, Hong Jiang

**Affiliations:** 1 Department of Neurology, Xiangya Hospital, Central South University, Changsha, Hunan, 410008, P. R. China; 2 State Key Laboratory of Medical Genetics of China, Central South University, Changsha, Hunan,410078, P. R. China; 3 Key Laboratory of Hunan Province in Neurodegenerative Disorders, Central South University, Changsha, Hunan, 410008, P. R. China; 4 School of Information Science and Engineering, Central South University, Hunan, 410083, P. R. China; 5 Hunan Engineering Laboratory for Advanced Control and Intelligent Automation, Hunan, 410083, P. R. China; 6 Department of Thoracic surgery, Xiangya Hospital, Central South University, Changsha, Hunan, 410008, P. R. China; 7 Department of Neurology at University of South China, The First People’s Hospital of Chenzhou, Chenzhou, Hunan, 423000, P. R. China; 8 Institute of Translational Medicine at University of South China, The First People’s Hospital of Chenzhou, Chenzhou, Hunan, 423000, P. R. China; Emory University, UNITED STATES

## Abstract

Autism spectrum disorder (ASD) comprise a group of neurodevelopmental disorders characterized by deficits in social and communication capacities and repetitive behaviors. Increasing neuroscientific evidence indicates that the neuropathology of ASD is widespread and involves epigenetic regulation in the brain. Differentially expressed miRNAs in the peripheral blood from autism patients were identified by high-throughput miRNA microarray analyses. Five of these miRNAs were confirmed through quantitative reverse transcription-polymerase chain reaction (qRT-PCR) analysis. A search for candidate target genes of the five confirmed miRNAs was performed through a Kyoto encyclopedia of genes and genomes (KEGG) biological pathways and Gene Ontology enrichment analysis of gene function to identify gene regulatory networks. To the best of our knowledge, this study provides the first global miRNA expression profile of ASD in China. The differentially expressed miR-34b may potentially explain the higher percentage of male ASD patients, and the aberrantly expressed miR-103a-3p may contribute to the abnormal ubiquitin-mediated proteolysis observed in ASD.

## Introduction

Autism spectrum disorder (ASD) comprise a group of chronic neurodevelopmental disorders characterized by social and language impairments and restricted and repetitive interests and behaviors [1]. ASD includes autistic disorder, Asperger's syndrome, and pervasive developmental disorder not otherwise specified (PDD-NOS)[2]. Patients with ASD vary greatly in terms of clinical manifestation and may also show associated medical comorbidities. Symptoms begin to appear at the age of three years, and affected individuals often require constant care from family members or professionals [3–5]. The Autism and Developmental Disabilities Monitoring (ADDM) Network calculated the prevalence of 8-year-old ASD as 14.7% based on the data from 11 ADDM Network sites in the USA obtained in 2010 [6]. The prevalence rates have increased significantly in the past decade, which may be due to the comprehensive effect of prenatal risk factors, environmental pollution, and heritable factors. It is one of the most heritable of the common neurodevelopmental diseases. Until now, it has been difficult to understand how diverse genetic susceptibilities translate to a clinical phenotype with so many genomic loci contributing to heterogeneous functions [7]. Thus far, studies have focused more on genetic factors, but only a few studies have investigated the role of miRNA in autism spectrum disorder [8–10].

MicroRNAs (miRNAs) are small endogenous non-coding regulatory RNAs (typically 21–23 nucleotides) that function as posttranscriptional regulators of gene expression [11, 12]. These are known to play a critical role in neurodevelopment, metabolism, neuroplasticity, apoptosis, and other fundamental neurobiological processes. By complementarily base-pairing with the 3’untranslated region (3’UTR) of specific target mRNAs, miRNA can regulate gene expression [13, 14]. Recent studies have demonstrated that miRNAs are present in human body fluids, such as serum, plasma, and cerebrospinal fluid. [15–18]. Several recent studies have reported differential miRNA expression in autism spectrum disorder. However, no specific study of regulatory miRNA expression and the gene regulatory networks of aberrant miRNAs has focused on autism spectrum disorder in China. Therefore, we performed the first investigation of the miRNA expression profile in the peripheral blood of Chinese autism patients and the gene regulatory networks of aberrant miRNAs.

## Materials and Methods

### Sample collection and RNA purification

5 autism patients (1 female and 4 males; mean age: 4.9±1.917) and 5 controls were recruited in microarray analysis study and another 15 patients (2 females and 13 males; mean age: 4.3±1.623) and 15 controls were recruited in qRT-PCR analysis study. The diagnosis of ASD was established in the patients with the use of the Diagnostic and Statistical Manual, Fourth Edition, Text Revision (DSM-IV-TR; American Psychiatric Association, 2000) criteria. Patients show typical ASD clinical symptoms, including deficits in communication and restricted patterns of interests or activities, while social deficits are observed in the school-age patients. Peripheral blood samples were obtained from participants (5ml blood for each) at Xiangya Hospital, and written informed consents were obtained from the parents of all of the participants because the participants were underage and did not have the capability to provide signed written informed consent. This study was approved by the Institutional Review Board and the Ethics Committee at Xiangya Hospital, Central South University (Changsha, China).

The total RNA was isolated using the Trizol method (Life Technologies, USA) according to the manufacturer’s instructions. The quality of RNA was examined using an Agilent Bioanalyzer (Santa Clara, CA, USA). The RNA quantity and purity were assessed using a K5500 micro-spectrophotometer (Kaiao, China). Values of A260/A28≥1.5 and A260/A230≥1 indicate acceptable RNA purity, and a value of RIN (RNA Integrity Number)≥7 obtained through the Agilent 2200 RNA assay indicates acceptable RNA integrity (Agilent, USA). Genomic DNA contamination was evaluated by gel electrophoresis. The total RNA was stored at -80°C until use.

### miRNA expression profiling by microarray

RiboArray miDETECT Human Array (A10101-1-12-19, 1×12K) microarrays were used to screen the miRNA expression profile (RIBOBIO, China). The microarrays contained 2,578 assay probes corresponding to the entire set of annotated human and nonhuman primate miRNA sequences from miRBase20.0. The internal control of the microarray assay was from RiboArray internal controls database referenced from multiple public probe database including Exiqon miRCURY LNA, GeneChip miRNA Array and Agilent miRNA. In this study, five autism patients aged 2.5 to 7 years of age and five age-matched controls were enrolled, and all of these were used as unique samples. For hybridization, 2.5 μg of total RNA labeled with Cy5 was used for each sample. To yield adequate microarray results, the labeling efficiency should be between 1.0 and 3.6. After overnight hybridization and washing, the fluorescence images were scanned using a GenePix 4000B laser scanner (Molecular Device, USA) and digitized using the R software. After normalization using the quantile normalization method [19], the *p*-values were calculated with the Rank Product Method [20]. miRNAs with p<0.05 were selected for cluster analysis. The raw data is available at Gene Expression Omnibus (accession number: GSE67979).

### miRNA qRT-PCR analysis

The total RNA was reverse transcribed to cDNA in a volume of 20 μL according to the manufacturer’s instructions. The five most differentially expressed miRNAs in the 15 autism patients and 15 health controls were screened for further validation by Bulge-Loop miRNA qRT-PCR assays (CFX96, Bio-Rad, USA). Has-miR-16-5p was used as an internal reference in the qRT-PCR experiments [15]. The miRNA qRT-PCR Primer Set (RiboBio Co. Guangzhou, China) was used to detect and quantify the expression of the five microRNAs (let-7a-5p, let-7d-5p, miR-34b-3p, miR-103a-3p, and miR-1228-3p) according to the manufacturer’s instructions [21]. Every qRT-PCR assay was performed in triplicate in a volume of 20 μL containing 1 μL of cDNA. The miRNA expression level was calculated using the 2^-ΔΔCt^ method [11].

### Investigation of gene regulatory networks based on miRNA expression

To validate the five most differentially expressed miRNAs validated by qRT-PCR analysis, several specific algorithms have been designed to predict the target genes of miRNAs. We first used four publicly available databases, namely TargetScan, miRanda, CLIP-Seq, and miRDB, for the prediction of potential target genes. Only those candidates that appeared in at least three databases were cinsidered candidate miRNA target genes. A Kyoto encyclopedia of genes and genomes (KEGG) biological pathway (http://www.genome.jp/) analysis and a Gene Ontology enrichment analysis of gene function were used to identify the gene networks that may be modulated by the dysregulated miRNAs. Subsequently, miRNA transcription factors were identified through Chip-Seq next-generation genome sequencing and based on data included in the ENCODE and GEO databases [22, 23]. Integrated miRNA regulatory networks were then constructed based on the above analysis.

### Statistical analysis

The microarray data were normalized using a quantile normalization method. The *p*-values were calculated with the Rank Product Method [19, 20], and differences with *p*< 0.01 were considered statistically significant. For analysis of the qRT-PCR results, a Wilcoxon rank-sum test was used, and differences with *p*< 0.05 was considered statistically significant [11]. Fisher’s exact test (*p*< 0.05) was used to identify significant biological pathways, and the false discovery rates (FDR) were used as significant corrections [24]. A hypergeometric distribution was used for the Gene Ontology enrichment analysis of gene function, and *p*< 0.01 was considered statistically significant [25].

## Result

### Microarray assay of dysregulated miRNA expression in autism spectrum disorder

The RNA extracted from the peripheral blood was analyzed using a RiboArray miDETECT Human Array. After normalization with the quantile normalization method, the hybridization data revealed 24 upregulated and 20 downregulated miRNAs (Table 1; more details in S1 Table). Interestingly, the dysregulated miR-15a and miR-15b previously discovered in autistic cerebellar cortices were found in this study to be also dysregulated in the peripheral blood [9]. miR-103, miR-92, and miR-19-2 have been reported to be aberrantly expressed in autistic lymphoblasts (Table 2), whereas the related miR-103a-3p, miR-92a-3p and miR-19b-3p were found to be abnormally expressed in autistic peripheral blood [8, 10, 26]. Thus, the differential expression of these miRNAs in the peripheral blood may partially reflect systemic changes in ASD.

**Table 1:** The differentially expressed miRNAs obtained from the microarray data^a^.

Up-regulated miRNAs					Down-regulated miRNAs				
miRNA ID	Accession number^b^	Mean ± SD		*p*-value	miRNA ID	Accession number^b^	Mean ± SD		*p*-value
		controls	patients				controls	patients	
hsa-miR-1273c	MIMAT0015017	10.815±1.807	12.326±1.528	0	hsa-miR-451a	MIMAT0001631	13.097±1.846	12.108±1.518	1.48E-05
hsa-miR-4299	MIMAT0016851	11.472±1.572	12.776±0.801	2.96E-05	hsa-miR-16-5p	MIMAT0000069	14.303±1.807	13.610±2.370	0.000751
hsa-miR-5739	MIMAT0023116	10.619±1.356	11.541±2.344	0.000336	hsa-miR-940	MIMAT0004983	12.604±0.781	11.832±1.196	0.000914
hsa-miR-6086	MIMAT0023711	12.118±0.749	13.092±0.393	0.000479	hsa-miR-574-3p	MIMAT0003239	13.543±1.213	12.967±1.083	0.001201
hsa-miR-494	MIMAT0002816	13.875±2.658	14.836±1.261	0.000687	hsa-let-7d-5p	MIMAT0000065	12.501±1.392	11.774±1.540	0.001215
hsa-miR-4270	MIMAT0016900	14.535±0.369	15.402±0.295	0.000805	hsa-let-7a-5p	MIMAT0000062	12.607±1.515	12.005±1.543	0.001448
hsa-miR-642a-3p	MIMAT0020924	11.565±0.856	12.497±0.394	0.00081	hsa-let-7f-5p	MIMAT0000067	11.268±1.840	10.931±1.573	0.001882
hsa-miR-4516	MIMAT0019053	12.816±2.250	13.213±2.188	0.001107	hsa-miR-92a-3p	MIMAT0000092	11.667±0.915	11.041±1.159	0.003172
hsa-miR-4436a	MIMAT0018952	10.036±0.814	10.981±0.136	0.001546	hsa-miR-3613-3p	MIMAT0017991	11.611±0.516	11.029±0.540	0.004125
hsa-miR-1246	MIMAT0005898	11.168±1.758	12.040±0.808	0.002055	hsa-miR-20a-5p	MIMAT0000075	12.332±1.069	11.799±0.993	0.004466
hsa-miR-575	MIMAT0003240	11.310±0.485	12.085±0.340	0.002821	hsa-miR-1228-3p	MIMAT0005583	12.391±1.506	12.095±1.673	0.005944
hsa-miR-4721	MIMAT0019835	11.715±0.259	12.376±0.544	0.003765	hsa-miR-3935	MIMAT0018350	14.062±1.749	13.863±1.483	0.006354
hsa-miR-483-5p	MIMAT0004761	11.676±1.349	12.407±0.485	0.004377	hsa-miR-4700-3p	MIMAT0019797	11.320±0.338	10.762±0.225	0.006453
hsa-miR-1249	MIMAT0005901	11.148±0.327	11.549±1.296	0.004407	hsa-miR-15b-5p	MIMAT0000417	11.050±1.161	10.733±1.234	0.006527
hsa-miR-4443	MIMAT0018961	9.865±0.774	10.524±0.494	0.004501	hsa-miR-15a-5p	MIMAT0000068	11.759±1.233	11.300±1.294	0.006942
hsa-miR-921	MIMAT0004971	10.094±0.352	10.745±0.427	0.005608	hsa-miR-4436b-5p	MIMAT0019940	11.854±0.924	11.438±0.585	0.007426
hsa-miR-34b-3p	MIMAT0004676	10.272±1.012	10.779±0.696	0.005909	hsa-miR-4665-5p	MIMAT0019739	11.620±1.406	11.521±1.133	0.007441
hsa-miR-6125	MIMAT0024598	13.084±2.164	13.256±1.945	0.006018	hsa-miR-19b-3p	MIMAT0000074	11.257±1.140	10.889±1.059	0.008103
hsa-miR-4669	MIMAT0019749	11.311±0.407	11.954±0.842	0.006897	hsa-miR-103a-3p	MIMAT0000101	12.390±1.097	11.965±1.118	0.009121
hsa-miR-34c-3p	MIMAT0004677	10.318±0.923	10.887±0.795	0.007624	hsa-miR-195-5p	MIMAT0000461	10.982±1.699	10.901±1.378	0.009279
hsa-miR-4728-5p	MIMAT0019849	11.756±0.617	12.280±0.495	0.008063					
hsa-miR-564	MIMAT0003228	10.076±0.278	10.679±0.497	0.00831					
hsa-miR-574-5p	MIMAT0004795	10.807±1.760	11.333±0.890	0.00873					
hsa-miR-4788	MIMAT0019958	12.324±0.978	12.940±0.483	0.009634					

a: More details are presented in S1 Table.

b: Accession numbers were obtained from miRBase database.

**Table 2:** The results of the present study *vs*. previous autism microRNA studies.

miRNA ID	Present result	Previous report	Type of sample	Reference
hsa-miR-16-5p	down-regulated	down-regulated	lymphoblastoid cell lines	Sarachana et al. [8]
hsa-miR-103	down-regulated	up-regulated	lymphoblastoid cell lines	Sarachana et al. [8]
hsa-miR-451	down-regulated	up-regulated	lymphoblastoid cell lines	Sarachana et al. [8]
miR-19b-3p	down-regulated	up-regulated	serum	Mundalil et al. [26]
miR-195-5p	down-regulated	up-regulated	serum	Mundalil et al. [26]
			lymphoblastoid cell lines	Sarachana et al. [8]
hsa-miR-15a	down-regulated	up-regulated	cerebellar cortex	Abu-Elneel et al. [9]
hsa-miR-15b	down-regulated	up-regulated	cerebellar cortex	Abu-Elneel et al. [9]
hsa-miR-92a-3p	down-regulated	down-regulated	lymphoblastoid cell lines	Talebizadeh et al. [10]

A subset of differentially expressed miRNAs was selected for clustering analysis. The correlation of the expression profiles between the biological replicates and treatment conditions was demonstrated through unsupervised hierarchical clustering analysis (S1 File).

### qRT-PCR confirmation of the expression of selected miRNAs

**Fig 1:**
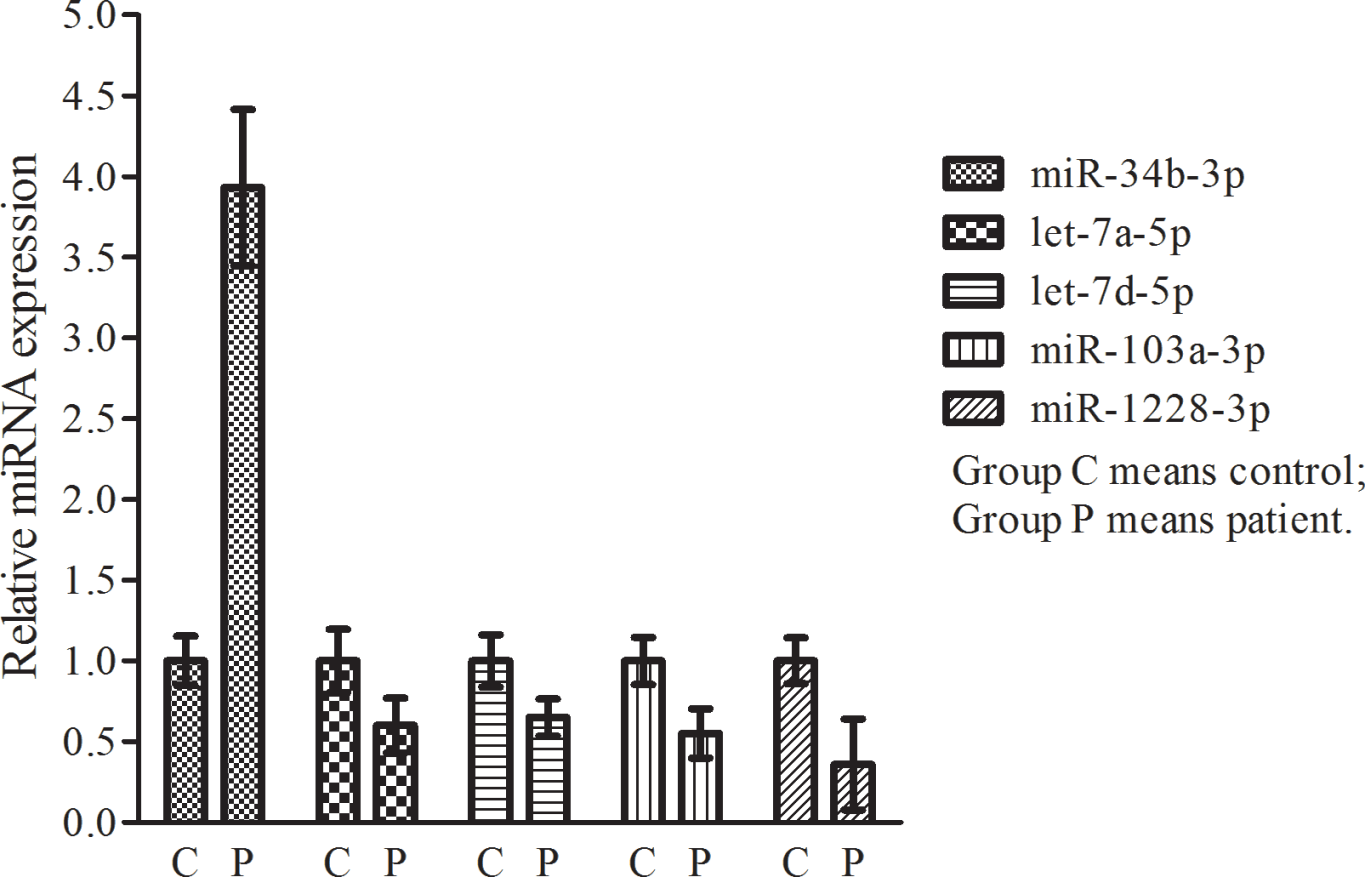
qRT-PCR confirmation of the expression of selected miRNAs. The levels of the five differentially expressed five miRNAs were statistically significant. The miR-34b expression level was significantly increased, whereas the levels of miR-let-7a, miR-let-7d, miR-103a, and miR-1228 were decreased.

Based on the dysregulated miRNA profiles obtained from the microarray analysis combined with recent research on the function of miRNAs, the five most aberrantly expressed miRNAs were selected for further qRT-PCR validation. As a result, the miR-34b expression level was significantly increased, whereas the levels of miR-let-7a, miR-let-7d, miR-103a, and miR-1228 were decreased (Fig 1, *p*< 0.05).

### Investigation of gene regulatory networks based on miRNA expression

**Fig 2:**
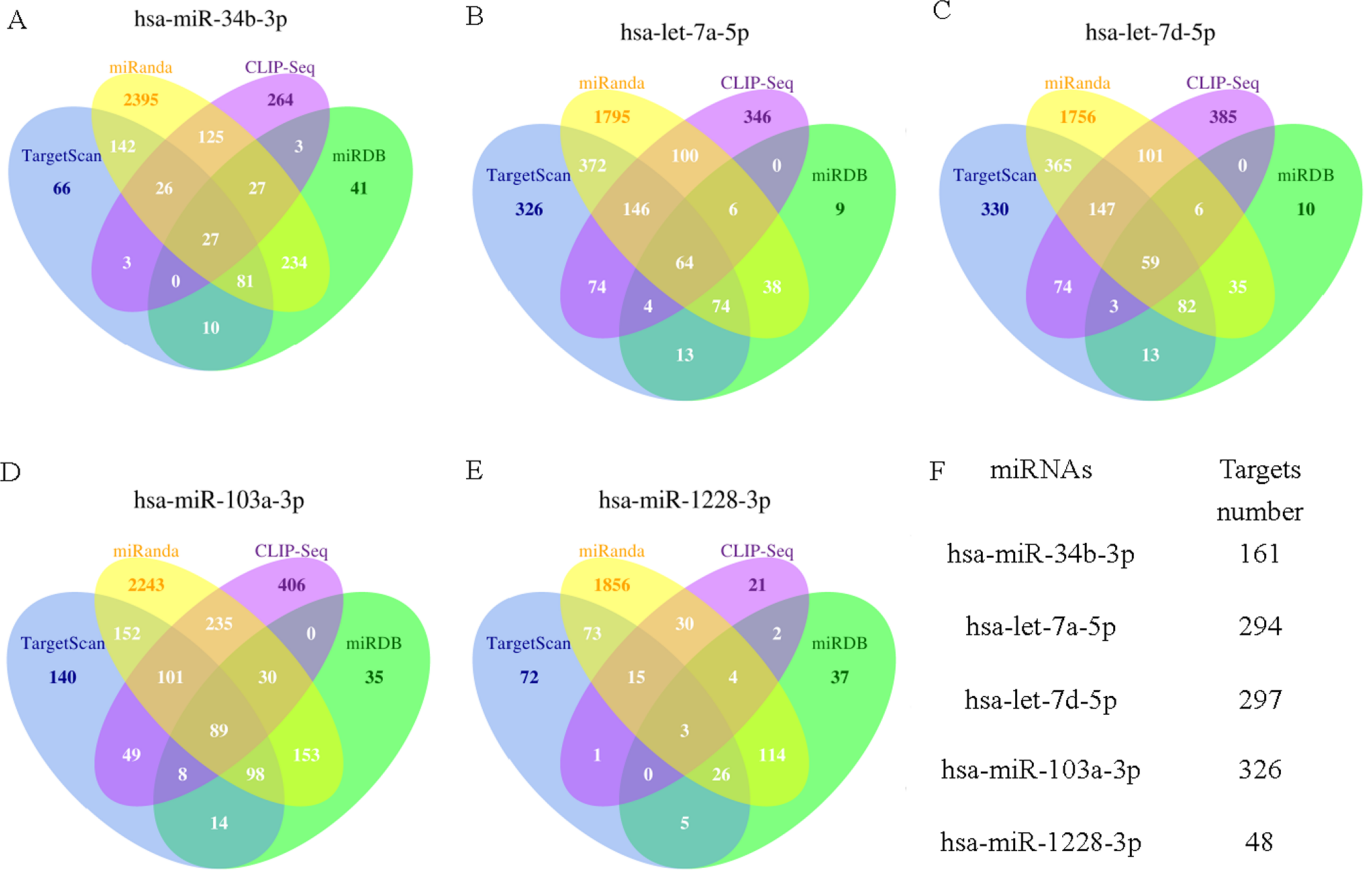
Prediction of target genes. Four databases were used for the prediction of target genes: TargetScan, miRanda, CLIP-Seq, and miRDB. Venn Diagrams A, B, C, D, and E show the intersection of each miRNA target gene predicted by various databases, and intersection of the target genes obtained from at least three databases. F indicates the total number of candidate target genes for each miRNA.

It has been predicted that the regulation of miRNAs is not a simple process. More than one target in different functional categories can be modulated by one miRNA, and more than one miRNA may target the same specific gene [10, 18]. Thus, the statistical data for the miRNA candidate target genes predicted by the four network databases (TargetScan, miRanda, CLIP-Seq, and miRDB) are presented in Fig 2. The Kyoto encyclopedia of genes and genomes (KEGG) biological pathway database was employed to extract the most relevant target genes on different biological pathways to predict the underlying connections among the target genes and their associated functions. The three most significant pathways for the five miRNAs were taken into account (Table 3). A Gene Ontology enrichment analysis of gene function was performed to fully explore the specific functions of the target genes. Interestingly, the results generated by the KEGG and GO analyses indicated correlations between the candidate targets of the miRNAs and autism and revealed other neurological functions previously and newly reported to be related to ASD, such as circadian rhythm-mammal [27], ubiquitin-mediated proteolysis (UPS) [28], dopaminergic synapse [29, 30], MAPK signaling pathway, development of primary male sexual characteristics and male sex differentiation in male gonad development.

**Table 3:** Kyoto encyclopedia of genes and genomes (KEGG) biological pathway analysis for the five miRNAs.

miRNA	Term	ID^a^	*P*-Value	Genes^b^
hsa-let-7a-5p	p53 signaling pathway	hsa04115	0.006623744	RRM2,MDM4,CDKN1A,CASP3,THBS1
	MAPK signaling pathway	hsa04010	0.00890767	NGF,CASP3,CHUK,MAP4K4,DUSP1,PLA2G3,DUSP4,
				NRAS,MAP4K3,GFBR1,PAK1
	TGF-beta signaling pathway	hsa04350	0.017204351	GDF6,E2F5,TGFBR1,THBS1,ACVR1C
hsa-let-7d-5p	Protein digestion and absorption	hsa04974	0.001653889	COL1A2,COL3A1,COL4A1,COL24A1,OL1A1,COL14A1,
				SLC16A10
	ECM-receptor interaction	hsa04512	0.007451271	COL1A2,COL3A1,COL4A1,COL24A1,THBS1,COL1A1
	MAPK signaling pathway	hsa04010	0.00776506	NGF,CASP3,CHUK,MAP4K4,DUSP1,PLA2G3,DUSP4,
				MAP4K3,NRAS,FAS,TGFBR1,PAK1
hsa-miR-34b-3p	Lysosome	hsa04142	0.003264022	LAMP1,AP3D1,GNS,M6PR,SCARB2
	RIG-I-like receptor signaling pathway	hsa04622	0.020271307	AZI2,DDX3X,CYLD
	Tight junction	hsa04530	0.024135052	MRAS,MLLT4,CTNNA1,CASK
hsa-miR-103a-3p	Ubiquitin mediated proteolysis	hsa04120	0.001387411	BTRC,FBXW7,UBE4A,HERC2,UBE2J1,CUL4A,WWP1,
				CDC23,UBE2Q1
	Circadian rhythm-mammal	hsa04710	0.002514562	NPAS2,PRKAB2,BTRC,CLOCK
	Dopaminergic synapse	hsa04728	0.00395313	KIF5A,PPP2R3C,KIF5C,ITPR1,SCN1A,CLOCK,PPP2R5C,PLCB1
hsa-miR-1228-3p	Adherens junction	hsa04520	0.012562355	PTPN1,TJP1
	Insulin signaling pathway	hsa04910	0.043243871	PTPN1,TSC1
	Dorso-ventral axis formation	hsa04320	0.05440918	ETV6

a: The ID numbers were obtained from the KEGG Pathway database.

b: The genes indicate the target genes in the pathway.

**Fig 3:**
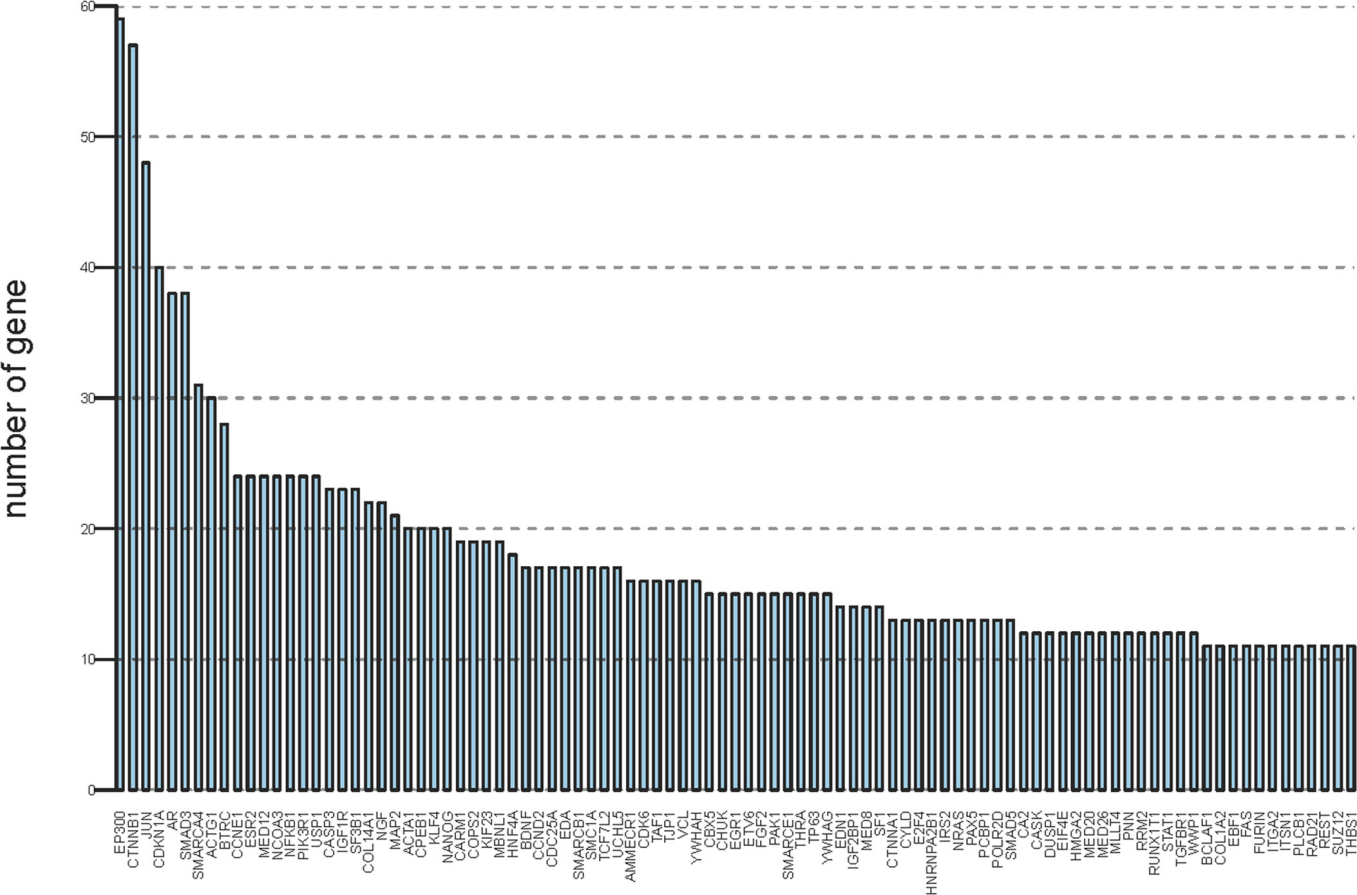
Importance of target genes and transcription factor genes. The horizontal axis indicates the names of target genes and transcription factor genes, and the vertical axis indicates the numbers of associated genes. The ranking was performed according to the number of associated genes, more than 10 of which could be shown.

We also included miRNA transcription factors in our study. Therefore, based on the above-described results, the S2 File shows the complex and integrated miRNA regulatory networks, which present the relationship between the miRNAs and mRNAs and the relationship between the miRNAs and miRNA transcription factors. Additionally, we calculated the number of associated genes for both candidate target genes and transcription factor genes in the miRNA regulatory interaction networks and ranked the number of associated genes, which was greater than 10 and is shown in Fig 3.

## Discussion

Autism spectrum disorder (ASD) is one of the most heritable neurodevelopmental diseases with a markedly increasing incidence in China. The characteristics of ASD include repetitive behaviors accompanying deficits in social interaction and communication [31]. Because the diagnosis of ASD mainly relies on psychometric evaluations, its diagnosis is often difficult and subjective. Therefore, molecular evidence for early and objective ASD diagnosis particularly non-invasive miRNA/gene biomarkers from the peripheral blood and genetic associations or interactions, is indispensable.

As described above, miRNAs are small non-coding regulatory RNAs that play an important role in fundamental neurobiological processes, particularly neurodevelopmental diseases. In Rett syndrome, multiple aberrantly expressed miRNAs, including miR-30a/d, miR-381, and miR-495, negatively regulate target gene translation [32]. In Down syndrome samples, five miRNAs (miR-155, miR-802, miR-125b-2, let-7c, and miR-99a) have been found to be overexpressed in fetal hippocampus and exclusively expressed in neurons [33]. In Fragile X syndrome, miR-367 negatively regulates *FXR1P* expression in human cell lines, and a loss of *FXR1P* downregulates brain-specific miRNAsthat are critical in neurodevelopment [34, 35].

A series of recent studies have shown that miRNAs in serum and plasma are considered biomarkers for several diseases [36]. Additionally, several studies have identified the expression profiles of miRNAs in ASD, and some of these studies presented data from lymphoblastoid cell cultures [5, 8, 10]. One of these studies included the analysis of samples from the cerebella of ASD patients [9]. We explored the miRNA expression profiles and the gene regulatory networks of aberrantly expressed miRNAs in autism spectrum disorder in China. In this study, five miRNAs (miR-103a, miR-34b, miR-let-7a, miR-let-7d, and miR-1228) were confirmed to be aberrantly expressed in the peripheral blood of ASD patients.

miR-34 modulates aging and neurodegeneration in *Drosophila* (dme-mir-34 and has-mir-34b are orthologous) [37], and miR-34b is elevated in spinocerebellar ataxia type 3/Machado-Joseph disease (SCA3/MJD) as demonstrated in our previous study [11]. These data illustrate the roles of miR-34b in both neurodevelopmental and neurodegenerative diseases. Mutiple results obtained in the current study indicate a relationship between miR-34b and ASD. The candidate target genes of miR-34b are mainly associated with central nervous system neuron development, long-term memory and tight junction pathways whose breakdown may result in diseases such as systemic inflammatory response syndrome (SIRS), inflammatory bowel disease, and autism [38]. Additionally, the strong association between the candidate target genes of miR-34b and ASD implies that miR-34b may play roles in the physiopathology of ASD. A linkage between autism and the *EIF4E* region on chromosome 4q has been found in genome-wide linkage studies [39], and functional variants modulating *EIF4E* expression have been previously indicated as risk factors for ASD [40]. Dysregulation of eukaryotic translation initiation factor 4E (eIF4E)-dependent translational control may contribute to ASD-like phenotypes [41]. *MAP2* encodes a protein that is enriched in dendrites and is implicated in determining and stabilizing dendritic shape during neuron development. Depleted *MAP2* neuronal expression and reduced dendrite numbers have been found in autistic adults [42]. In addition, the *MAP2* gene has been identified a good candidate for the generation of a preserved speech variant (PSV) in a patient with autism and Rett-like features [43]. Interestingly, the candidate target genes of miR-34b also affect the development of primary male sexual characteristics, male sex differentiation and male gonad development, which may explain the higher percentage of males in the ASD group.

miR-103 and miR-107 have been reported to be highly expressed in brain tissue [44]. Sarachana et al. previously hypothesized that the dysregulation of miR-103/7 may result in abnormal lipid and fatty acid metabolism in ASD [8]. Several findings of our study provide plausible evidence that miR-103a-3p plays a role in the physiopathological mechanism of ASD. The candidate target genes of miR-103a-3p reveal close relationships with the dysfunctional physiological pathways related to ASD, such as central nervous system development, neuron projection morphogenesis and skeletal muscle tissue and organ development, and with various pathways, including ubiquitin-mediated proteolysis (UPS), circadian rhythm-mammal [8, 26] and dopaminergic synapse [28, 29]. Directly, HECT-type E3 ubiquitin ligase (UBE3A) dysfunction appears to induce a large spectrum of pediatric cognitive dysfunctions, such as autism and Rett and Angelman syndromes, depending on the mechanism and severity of UBE3A loss [27]. Indirectly, UPS is involved in neural functions and co-morbid diseases with ASD, such as synaptic dysfunctions and muscle diseases. Ubiquitination is a highly specific method for manipulating protein expression at the neural synapse [45, 46] and overloading or other perturbations of ubiquitin-dependent proteolysis is likely involved in inclusion myopathies [27]. Based on the results of various previous studies [8], we hypothesized that the aberrantly expressed miR-103a-3p may contribute to abnormal ubiquitin-mediated proteolysis in ASD. Specifically, the candidate target gene *BTRC* is involved not only in the mammal circadian rhythm and mammal and ubiquitin-mediated proteolysis but also in the Wnt signaling pathway [47], all of which have been acknowledged to play roles in ASD. Additionally, one candidate target gene of miR-103a-3p, *BDNF*, encodes brain-derived neurotrophic factor (BDNF). Increasing evidence suggests a direct or indirect involvement of BDNF in autism [48], and this factor plays a key role in neuronal survival, morphology, differentiation and synaptic strength [49]. It is increased in some ASD patients as well as in some animal models of ASD [50, 51].

Other candidate miRNAs have also associated with neuronal physiology and pathology. A previous study suggested that embryonic development may be relevant to ASD [8]. In addition, miR-let-7a expression have been found to be high in early human embryonic tissues and abruptly decreases thereafter, which suggests that miR-let-7a plays critical roles in early embryonic development [52]. Therefore, miR-let-7a may be indirectly associated with ASD through the embryonic development process. miR-let-7d suppresses neural stem cell proliferation by reducing *TLX* expression in neural stem cells, which may be a novel strategy for identifying potential interventions in relevant neurological diseases [53]. Notably, the candidate target genes of both miR-let-7a and miR-let-7d are associated with the MAPK signaling pathway, which directly or indirectly play roles in the physiopathology of ASD. Ghahramani Seno et al. previously suggested that mitogen-activated protein kinases (MAPK) may be involved in the development of autism [5]. In addition, several genes in the MAPK signaling pathway have been reported to be relevant to ASD. *NGF* encodes nerve growth factor (NGF) and influences inflammatory responses and neuron projection development. The serum NGF concentrations have been reported to be significantly higher in autistic children [54]. Another analysis suggests that *NGF* is a potential risk gene for nonverbal communication (NVC) impairments [55]. Recent studies have implied an association between mitochondrial dysfunction and ASD [56]. The protein levels of caspase-3, which is encoded by *CASP3*, are also increased in ASD patients [57]. *NRAS* plays roles in the regulation of long-term neuronal synaptic plasticity and transmission and is considered a candidate gene for autism. This finding may provide a novel insight into the etiology of autism [58].

Interestingly, the candidate target genes of miR-1228-3p were found to not be involved in brain-specific or brain-related functions but involved in the process of hepatocyte growth factor (HGF) production. A recent study reported that decreased HGF levels are found in autistic children and suggested that the serum HGF concentration may be a useful biomarker of autistic children [59]. Additionally, our findings show an association between the miR-1228-3p target genes and the insulin signaling pathway, which are hypothesized to contribute to the development of autism in genetically susceptible individuals by promoting PI3K/Tor pathway activation in neurons [60].

Taken together, four of the five aberrantly expressed miRNAs are related to the brain, suggesting that the gene expression differences observed in the peripheral blood may indicate similar alterations in the brain that are likely attribute to a system-scale differential expression of miRNAs.

## Conclusion

In summary, we investigated the global miRNA expression profiles of ASD in China, confirming that the use of alternative and more easily accessible peripheral blood for the study of miRNA expression in ASD is valid. The differentially expressed miR-34b may potentially explain why ASD is markedly more common in males than in females. The aberrantly expressed miR-103a-3p may contribute to abnormal ubiquitin-mediated proteolysis in ASD. Thus, these five miRNAs are beneficial for better understanding ASD-specific miRNA expression profiles in peripheral blood and provide new insights into future researches for ASD.

## Supporting Information

S1 Table More details of microarray analysis data. (XLSX)

S1 File Hierarchical cluster analysis. An unsupervised hierarchical cluster analysis of 150 significantly differentially expressed miRNAs among all of the autistic individuals (A6, A357, A14, A15, A16) and controls (4, 567, 28, 29, 30) shows the distinct miRNA expression pattern of the two groups (*p* < 0.05).
(PDF)

S2 File Complex and integrated miRNA regulatory networks. The image shows not only the relationship between miRNAs and mRNAs or transcriptional factors (TFs) but also the interactive relationships between the proteins of the target genes. Orange rhombi: miRNAs, blue circles: mRNAs, aubergine circles: TFs, pink lines: miRNA-mRNA interactions, blue lines: mRNA-TF interactions, green lines: miRNA-TF interactions.
(PDF)

## References

[1] Kolevzon A, Gross R, Reichenberg A (2007) Prenatal and perinatal risk factors for autism: a review and integration of findings. Arch Pediatr Adolesc Med 161(4): 326–333. 17404128 10.1001/archpedi.161.4.326

[2] Dawson S, Glasson EJ, Dixon G, Bower C (2009) Birth defects in children with autism spectrum disorders: a population-based, nested case-control study. Am J Epidemiol 169(11): 1296–1303. 10.1093/aje/kwp059 19372213

[3] Goyal DK, Miyan JA (2014) Neuro-immune abnormalities in autism and their relationship with the environment: a variable insult model for autism. Front Endocrinol (Lausanne) 5: 29 10.3389/fendo.2014.00029 24639668 PMC3945747

[4] DeJong H, Bunton P, Hare DJ (2014) A systematic review of interventions used to treat catatonic symptoms in people with autistic spectrum disorders. J Autism Dev Disord 44(9): 2127–2136. 10.1007/s10803-014-2085-y 24643578

[5] Ghahramani Seno MM, Hu P, Gwadry FG, Pinto D, Marshall CR, Casallo G, et al (2011) Gene and miRNA expression profiles in autism spectrum disorders. Brain Res 1380: 85–97. 10.1016/j.brainres.2010.09.046 20868653

[6] Wingate M, Kirby RS, Pettygrove S, Cunniff C, Schulz E, Ghosh T, et al (2014) Prevalence of autism spectrum disorder among children aged 8 years—autism and developmental disabilities monitoring network, 11 sites, United States, 2010. MMWR Surveill Summ 63(2): 1–21. 24670961

[7] Holt R, Monaco AP (2011) Links between genetics and pathophysiology in the autism spectrum disorders. EMBO Mol Med 3(8): 438–450. 10.1002/emmm.201100157 21805639 PMC3377085

[8] Sarachana T, Zhou R, Chen G, Manji HK, Hu VW (2010) Investigation of post-transcriptional gene regulatory networks associated with autism spectrum disorders by microRNA expression profiling of lymphoblastoid cell lines. Genome Med 2(4): 23 10.1186/gm144 20374639 PMC2873801

[9] Abu-Elneel K, Liu T, Gazzaniga FS, Nishimura Y, Wall DP, Geschwind DH, et al (2008) Heterogeneous dysregulation of microRNAs across the autism spectrum. Neurogenetics 9(3): 153–161. 10.1007/s10048-008-0133-5 18563458

[10] Talebizadeh Z, Butler MG, Theodoro MF (2008) Feasibility and relevance of examining lymphoblastoid cell lines to study role of microRNAs in autism. Autism Res 1(4): 240–250. 10.1002/aur.33 19360674 PMC2768334

[11] Shi Y, Huang F, Tang B, Li J, Wang J, Shen L, et al (2014) MicroRNA profiling in the serums of SCA3/MJD patients. Int J Neurosci 124(2): 97–101. 10.3109/00207454.2013.827679 23879331

[12] Bartel DP (2009) MicroRNAs: target recognition and regulatory functions. Cell 136(2): 215–233. 10.1016/j.cell.2009.01.002 19167326 PMC3794896

[13] Aw S, Cohen SM (2012) Time is of the essence: microRNAs and age-associated neurodegeneration. Cell Res 22(8): 1218–1220. 10.1038/cr.2012.59 22491478 PMC3411169

[14] Krol J, Loedige I, Filipowicz W (2010) The widespread regulation of microRNA biogenesis, function and decay. Nat Rev Genet 11(9): 597–610. 10.1038/nrg2843 20661255

[15] Huang Z, Huang D, Ni S, Peng Z, Sheng W, Du X (2010) Plasma microRNAs are promising novel biomarkers for early detection of colorectal cancer. Int J Cancer 127(1): 118–126. 10.1002/ijc.25007 19876917

[16] Cogswell JP, Ward J, Taylor IA, Waters M, Shi Y, Cannon B, et al (2008) Identification of miRNA changes in Alzheimer's disease brain and CSF yields putative biomarkers and insights into disease pathways. J Alzheimers Dis 14(1): 27–41. 18525125 10.3233/jad-2008-14103

[17] Gilad S, Meiri E, Yogev Y, Benjamin S, Lebanony D, Yerushalmi N, et al (2008) Serum microRNAs are promising novel biomarkers. PLoS One 3(9): e3148 10.1371/journal.pone.0003148 18773077 PMC2519789

[18] Sevcikova S, Kubiczkova L, Sedlarikova L, Slaby O, Hajek R (2013) Serum miR-29a as a marker of multiple myeloma. Leuk Lymphoma 54(1): 189–191. 10.3109/10428194.2012.704030 22712836

[19] Bolstad BM, Irizarry RA, Astrand M, Speed TP (2003) A comparison of normalization methods for high density oligonucleotide array data based on variance and bias. Bioinformatics 19(2): 185–193. 12538238 10.1093/bioinformatics/19.2.185

[20] Hong F, Breitling R, McEntee CW, Wittner BS, Nemhauser JL, Chory J (2006) RankProd: a bioconductor package for detecting differentially expressed genes in meta-analysis. Bioinformatics 22(22): 2825–2827. 16982708 10.1093/bioinformatics/btl476

[21] Li YQ, Zhang MF, Wen HY, Hu CL, Liu R, Wei HY, et al (2013) Comparing the diagnostic values of circulating microRNAs and cardiac troponin T in patients with acute myocardial infarction. Clinics (Sao Paulo) 68(1): 75–80. 23420161 10.6061/clinics/2013(01)OA12 PMC3552456

[22] Myers RM, Stamatoyannopoulos J, Snyder M, Dunham I, Hardison RC, Bernstein BE, et al (2011) A user's guide to the encyclopedia of DNA elements (ENCODE). PLoS Biol 9(4): e1001046 10.1371/journal.pbio.1001046 21526222 PMC3079585

[23] Arvey A, Agius P, Noble WS, Leslie C (2012) Sequence and chromatin determinants of cell-type-specific transcription factor binding. Genome Res 22(9): 1723–1734. 10.1101/gr.127712.111 22955984 PMC3431489

[24] Kanehisa M, Goto S (2000) KEGG: kyoto encyclopedia of genes and genomes. Nucleic Acids Res 28(1): 27–30. 10592173 10.1093/nar/28.1.27 PMC102409

[25] Rivals I, Personnaz L, Taing L, Potier MC (2007) Enrichment or depletion of a GO category within a class of genes: which test? Bioinformatics 23(4): 401–407. 17182697 10.1093/bioinformatics/btl633

[26] Mundalil Vasu M, Anitha A, Thanseem I, Suzuki K, Yamada K, Takahashi T, et al (2014) Serum microRNA profiles in children with autism. Mol Autism 5: 40 10.1186/2040-2392-5-40 25126405 PMC4132421

[27] Corbett BA, Mendoza S, Abdullah M, Wegelin JA, Levine S (2006) Cortisol circadian rhythms and response to stress in children with autism. Psychoneuroendocrinology 31(1): 59–68. 16005570 10.1016/j.psyneuen.2005.05.011

[28] Lehman NL (2009) The ubiquitin proteasome system in neuropathology. Acta Neuropathol 118(3): 329–347. 10.1007/s00401-009-0560-x 19597829 PMC2716447

[29] Karayannis T, Au E, Patel JC, Kruglikov I, Markx S, Delorme R, et al (2014) Cntnap4 differentially contributes to GABAergic and dopaminergic synaptic transmission. Nature 511(7508): 236–240. 24870235 10.1038/nature13248 PMC4281262

[30] Penzes P, Buonanno A, Passafaro M, Sala C, Sweet RA (2013) Developmental vulnerability of synapses and circuits associated with neuropsychiatric disorders. J Neurochem 126(2): 165–182. 10.1111/jnc.12261 23574039 PMC3700683

[31] Singh AS, Chandra R, Guhathakurta S, Sinha S, Chatterjee A, Ahmed S, et al (2013) Genetic association and gene-gene interaction analyses suggest likely involvement of ITGB3 and TPH2 with autism spectrum disorder (ASD) in the Indian population. Prog Neuropsychopharmacol Biol Psychiatry 45: 131–143. 10.1016/j.pnpbp.2013.04.015 23628433

[32] Wu H, Tao J, Chen PJ, Shahab A, Ge W, Hart RP, et al (2010) Genome-wide analysis reveals methyl-CpG-binding protein 2-dependent regulation of microRNAs in a mouse model of Rett syndrome. Proc Natl Acad Sci U S A 107(42): 18161–18166. 10.1073/pnas.1005595107 20921386 PMC2964235

[33] Kuhn DE, Nuovo GJ, Martin MM, Malana GE, Pleister AP, Jiang J, et al (2008) Human chromosome 21-derived miRNAs are overexpressed in down syndrome brains and hearts. Biochem Biophys Res Commun 370(3): 473–477. 10.1016/j.bbrc.2008.03.120 18387358 PMC2585520

[34] Cheever A, Blackwell E, Ceman S (2010) Fragile X protein family member FXR1P is regulated by microRNAs. Rna 16(8): 1530–1539. 10.1261/rna.2022210 20519410 PMC2905753

[35] Xu XL, Zong R, Li Z, Biswas MH, Fang Z, Nelson DL, et al (2011) FXR1P but not FMRP regulates the levels of mammalian brain-specific microRNA-9 and microRNA-124. J Neurosci 31(39): 13705–13709. 10.1523/JNEUROSCI.2827-11.2011 21957233 PMC3446782

[36] Scholer N, Langer C, Dohner H, Buske C, Kuchenbauer F (2010) Serum microRNAs as a novel class of biomarkers: a comprehensive review of the literature. Exp Hematol 38(12): 1126–1130. 10.1016/j.exphem.2010.10.004 20977925

[37] Liu N, Landreh M, Cao K, Abe M, Hendriks GJ, Kennerdell JR, et al (2012) The microRNA miR-34 modulates ageing and neurodegeneration in Drosophila. Nature 482(7386): 519–523. 10.1038/nature10810 22343898 PMC3326599

[38] Liu Z, Li N, Neu J (2005) Tight junctions, leaky intestines, and pediatric diseases. Acta Paediatr 94(4): 386–393. 16092447 10.1111/j.1651-2227.2005.tb01904.x

[39] Neves-Pereira M, Muller B, Massie D, Williams JH, O'Brien PC, Hughes A, et al (2009) Deregulation of EIF4E: a novel mechanism for autism. J Med Genet 46(11): 759–765. 10.1136/jmg.2009.066852 19556253

[40] Waltes R, Gfesser J, Haslinger D, Schneider-Momm K, Biscaldi M, Voran A, et al (2014) Common EIF4E variants modulate risk for autism spectrum disorders in the high-functioning range. J Neural Transm 121(9): 1107–1116. 10.1007/s00702-014-1230-2 24818597

[41] Gkogkas CG, Khoutorsky A, Ran I, Rampakakis E, Nevarko T, Weatherill DB, et al (2013) Autism-related deficits via dysregulated eIF4E-dependent translational control. Nature 493(7432): 371–377. 10.1038/nature11628 23172145 PMC4133997

[42] Mukaetova-Ladinska EB, Arnold H, Jaros E, Perry R, Perry E (2004) Depletion of MAP2 expression and laminar cytoarchitectonic changes in dorsolateral prefrontal cortex in adult autistic individuals. Neuropathol Appl Neurobiol 30(6): 615–623. 15541002 10.1111/j.1365-2990.2004.00574.x

[43] Pescucci C, Meloni I, Bruttini M, Ariani F, Longo I, Mari F, et al (2003) Chromosome 2 deletion encompassing the MAP2 gene in a patient with autism and Rett-like features. Clin Genet 64(6): 497–501. 14986829 10.1046/j.1399-0004.2003.00176.x

[44] Wilfred BR, Wang WX, Nelson PT (2007) Energizing miRNA research: a review of the role of miRNAs in lipid metabolism, with a prediction that miR-103/107 regulates human metabolic pathways. Mol Genet Metab 91(3): 209–217. 17521938 10.1016/j.ymgme.2007.03.011 PMC1978064

[45] Schwartz AL, Ciechanover A (2009) Targeting proteins for destruction by the ubiquitin system: implications for human pathobiology. Annu Rev Pharmacol Toxicol 49: 73–96. 10.1146/annurev.pharmtox.051208.165340 18834306

[46] Yi JJ, Ehlers MD (2005) Ubiquitin and protein turnover in synapse function. Neuron 47(5): 629–632. 16129392 10.1016/j.neuron.2005.07.008

[47] Martin PM, Yang X, Robin N, Lam E, Rabinowitz JS, Erdman CA, et al (2013) A rare WNT1 missense variant overrepresented in ASD leads to increased Wnt signal pathway activation. Transl Psychiatry 3: e301 10.1038/tp.2013.75 24002087 PMC3784764

[48] Halepoto DM, Bashir S, L ALA (2014) Possible role of brain-derived neurotrophic factor (BDNF) in autism spectrum disorder: current status. J Coll Physicians Surg Pak 24(4): 274–278. 24709243

[49] Binder DK, Scharfman HE (2004) Brain-derived neurotrophic factor. Growth Factors 22(3): 123–131. 15518235 10.1080/08977190410001723308 PMC2504526

[50] Koh JY, Lim JS, Byun HR, Yoo MH (2014) Abnormalities in the zinc-metalloprotease-BDNF axis may contribute to megalencephaly and cortical hyperconnectivity in young autism spectrum disorder patients. Mol Brain 7: 64 10.1186/s13041-014-0064-z 25182223 PMC4237964

[51] Connolly AM, Chez M, Streif EM, Keeling RM, Golumbek PT, Kwon JM, et al (2006) Brain-derived neurotrophic factor and autoantibodies to neural antigens in sera of children with autistic spectrum disorders, Landau-Kleffner syndrome, and epilepsy. Biol Psychiatry 59(4): 354–363. 16181614 10.1016/j.biopsych.2005.07.004

[52] Lozoya T, Dominguez F, Romero-Ruiz A, Steffani L, Martinez S, Monterde M, et al (2014) The Lin28/Let-7 system in early human embryonic tissue and ectopic pregnancy. PLoS One 9(1): e87698 10.1371/journal.pone.0087698 24498170 PMC3909210

[53] Zhao C, Sun G, Ye P, Li S, Shi Y (2013) MicroRNA let-7d regulates the TLX/microRNA-9 cascade to control neural cell fate and neurogenesis. Sci Rep 3: 1329 10.1038/srep01329 23435502 PMC3580325

[54] Dincel N, Unalp A, Kutlu A, Ozturk A, Uran N, Ulusoy S (2013) Serum nerve growth factor levels in autistic children in Turkish population: a preliminary study. Indian J Med Res 138(6): 900–903. 24521633 PMC3978979

[55] Lu AT, Yoon J, Geschwind DH, Cantor RM (2013) QTL replication and targeted association highlight the nerve growth factor gene for nonverbal communication deficits in autism spectrum disorders. Mol Psychiatry 18(2): 226–235. 10.1038/mp.2011.155 22105621 PMC3586745

[56] Rossignol DA, Frye RE (2012) A review of research trends in physiological abnormalities in autism spectrum disorders: immune dysregulation, inflammation, oxidative stress, mitochondrial dysfunction and environmental toxicant exposures. Mol Psychiatry 17(4): 389–401. 10.1038/mp.2011.165 22143005 PMC3317062

[57] Siniscalco D, Sapone A, Giordano C, Cirillo A, de Novellis V, de Magistris L, et al (2012) The expression of caspases is enhanced in peripheral blood mononuclear cells of autism spectrum disorder patients. J Autism Dev Disord 42(7): 1403–1410. 10.1007/s10803-011-1373-z 21969075

[58] Xia K, Guo H, Hu Z, Xun G, Zuo L, Peng Y, et al (2014) Common genetic variants on 1p13.2 associate with risk of autism. Mol Psychiatry 19(11): 1212–1219. 10.1038/mp.2013.146 24189344

[59] Russo AJ, Krigsman A, Jepson B, Wakefield A (2009) Decreased Serum Hepatocyte Growth Factor (HGF) in Autistic Children with Severe Gastrointestinal Disease. Biomark Insights 4: 181–190. 20029653 10.4137/bmi.s3656 PMC2796865

[60] Stern M (2011) Insulin signaling and autism. Front Endocrinol (Lausanne) 2: 54 10.3389/fendo.2011.00054 22649376 PMC3355926

