# Supplementary figures and images for: Investigation of Gene Regulatory Networks Associated with Autism Spectrum Disorder Based on MiRNA Expression in China
Source: PLoS One. 2015 Jun 10;10(6):e0129052. doi: 10.1371/journal.pone.0129052 (PMC4462583; doi:10.1371/journal.pone.0129052)

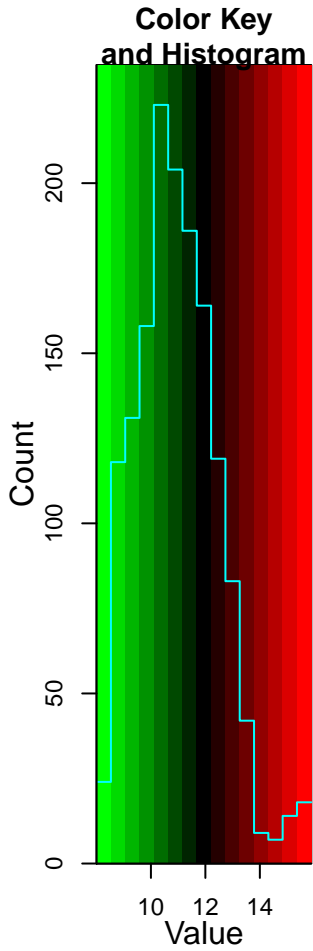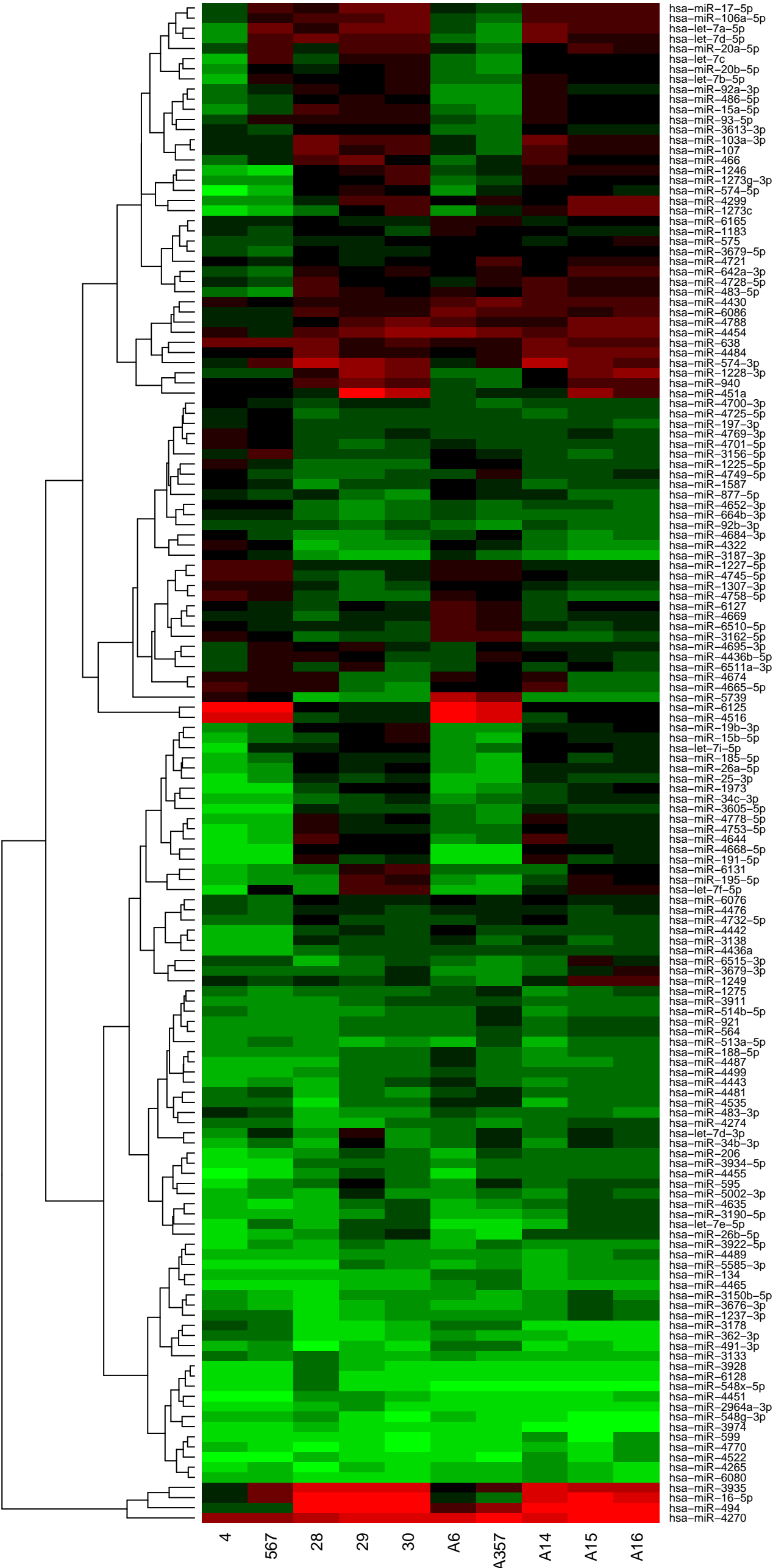

Supplement: S1 File — An unsupervised hierarchical cluster analysis of 150 significantly differentially expressed miRNAs among all of the autistic individuals (A6, A357, A14, A15, A16) and controls (4, 567, 28, 29, 30) shows the distinct miRNA expression pattern of the two groups (p < 0.05). (PDF) [file pone.0129052.s002.pdf]

hsa-miR-34b-3p

hsa-miR-103a-3p

hsa-let-7d-5p

hsa-let-7a-5p

hsa-miR-1228-3p

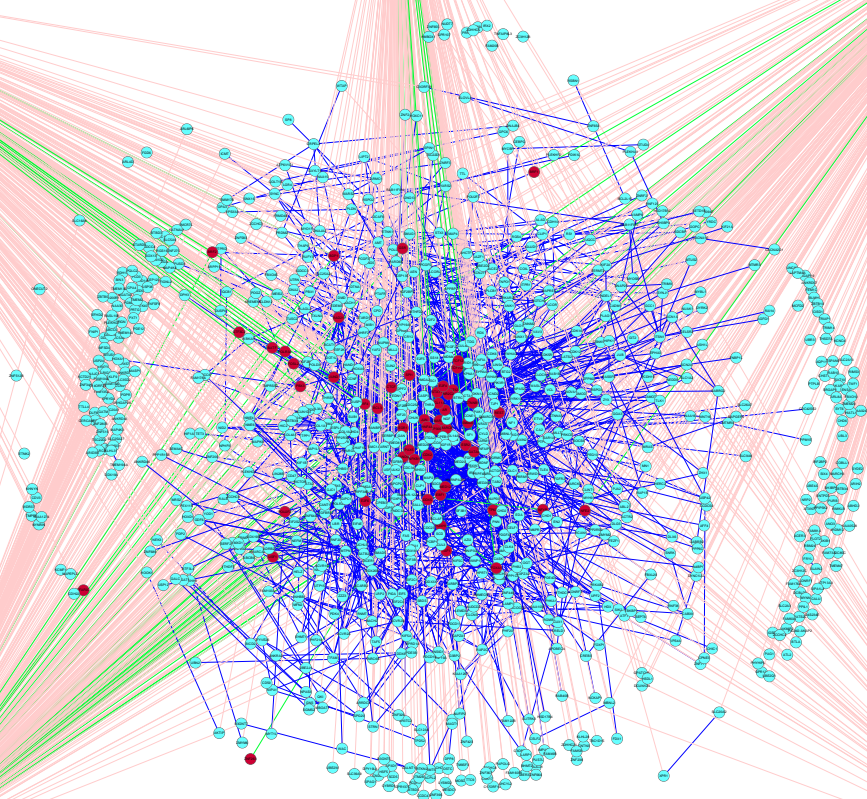

Supplement: S2 File — The image shows not only the relationship between miRNAs and mRNAs or transcriptional factors (TFs) but also the interactive relationships between the proteins of the target genes. Orange rhombi: miRNAs, blue circles: mRNAs, aubergine circles: TFs, pink lines: miRNA-mRNA interactions, blue lines: mRNA-TF interactions, green lines: miRNA-TF interactions. (PDF) [file pone.0129052.s003.pdf]
